# Schwann cell reprogramming via EMT-like program following peripheral nerve injury and during nerve regeneration

**DOI:** 10.3389/fcell.2025.1621380

**Published:** 2025-07-09

**Authors:** Wenyu Dai, Yang Miao, Sheng Yi

**Affiliations:** ^1^ Key Laboratory of Neuroregeneration of Jiangsu and Ministry of Education, Co-Innovation Center of Neuroregeneration, Nantong University, Nantong, Jiangsu, China; ^2^ Department of Pharmacy, The First People’s Hospital of Yancheng, The Yancheng Clinical College of Xuzhou Medical University, Yancheng, Jiangsu, China

**Keywords:** Schwann cells, mesenchymal-epithelial transition-like program, peripheral nerve injury, cell plasticity, nerve regeneration

## Abstract

The activation of the epithelial-mesenchymal transition (EMT) enhances cell plasticity and plays a pivotal role in driving critical biological processes such as embryonic process, tissue repair, and cancer metastasis. EMT is regulated by multiple signaling pathways, including transforming growth factor-β (TGF-β), Wnt, and Notch signaling, and is finely orchestrated by a network of transcriptional factors, epigenetic modifications (such as DNA methylation and histone alterations), and non-coding RNAs. In the peripheral nervous system, Schwann cells undergo a distinct EMT-like transformation following nerve injury, adopting a repair phenotype known as repair Schwann cells. These repair Schwann cells play a multifaceted role in nerve regeneration by clearing myelin debris, secreting regeneration-promoting factors, mediating structural reorganization, and creating a conducive microenvironment for axonal regrowth. Therapeutic strategies targeting the regulation of the EMT-like program of Schwann cells thus hold significant promise for the treatment of peripheral nerve injury, particularly in cases of severe nerve injury with incomplete recovery and poor functional restoration.

## Introduction

**FIGURE 1:**
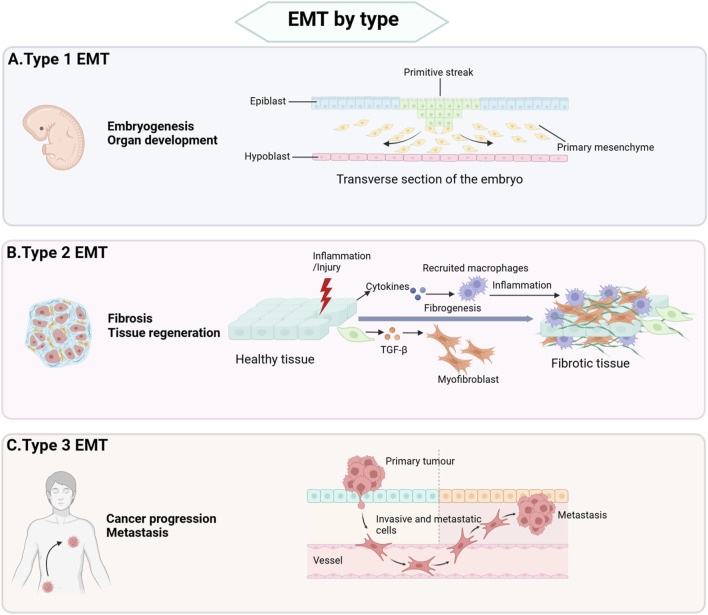
Types of EMT. **(A)** Type 1 EMT is primarily associated with critical developmental processes, including implantation, embryogenesis, and organ formation. **(B)** Type 2 EMT plays a central role in post-injury repair mechanisms, such as wound healing, tissue regeneration, and the pathogenesis of organ fibrosis. This type of EMT is essential for restoring tissue integrity and function following damage. **(C)** Type 3 EMT is closely linked tumor cell invasion and metastasis and participates in cancer progression.

The epithelial-mesenchymal transition (EMT) is a biological process in which epithelial cells lose their cellular polarity and transform into motile mesenchymal cells. During EMT, cells switch from a tightly packed epithelial state characterized by apical-basal polarity to a mesenchymal state that exhibits enhanced migratory and invasive capabilities. EMT plays a critical role in various physiological and pathological contexts, including embryonic development, wound healing, tissue regeneration, fibrosis, and cancer progression. Based on distinct biological functions and outcomes, EMT is commonly divided to three types named as type 1 EMT, type 2 EMT, and type 3 EMT (Figure 1).

Type 1 EMT is primarily involved in implantation, embryogenesis, and organ development. During embryonic development, primitive epithelial cells undergo type 1 EMT to form primary mesenchyme. Subsequently, primary mesenchyme can revert to secondary epithelia through a reverse process of EMT known as mesenchymal-epithelial transition (MET), ultimately generating connective tissue cells (Chen et al., 2017). In contrast, type 2 and type 3 EMT are associated with pathogenic processes. Type 2 EMT contributes to wound healing, tissue regeneration, and organ fibrosis, whereas type 3 EMT drives tumor cell invasion and metastasis (Kalluri and Weinberg, 2009).

Peripheral nerve injury represents a significant clinical challenge, with an annual incidence rate of 13–23 cases per 100,000 individuals in developed countries (Li et al., 2014). Injury to peripheral nerves disrupts the critical signal transmission between the central nervous system and the rest of the body, impairing neural communication with muscles, skin, and organs, ultimately leading to compromised autonomic, motor, and sensory functions. Unlike injured central nerves, peripheral nerves possess a remarkable intrinsic regenerative capacity. Schwann cells critically facilitate the regeneration process following peripheral nerve injury.

**FIGURE 2:**
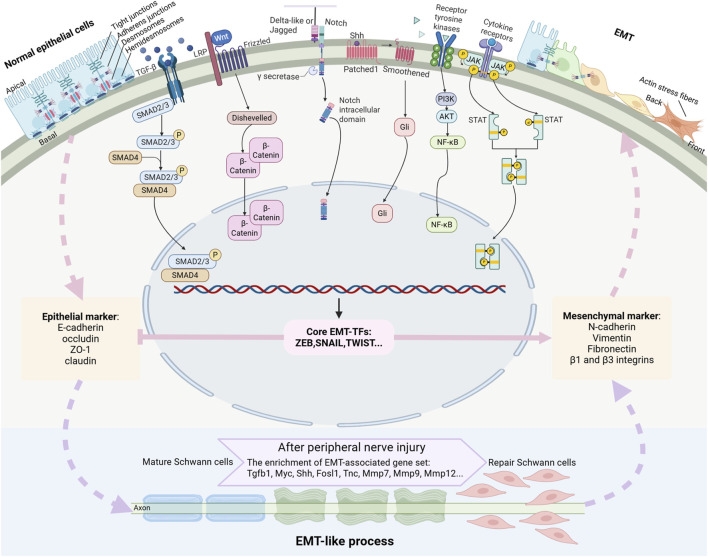
Outline of a typical EMT program and the signaling pathways driving EMT. EMT is a highly dynamic process that mediates the transformation of epithelial cells characterized by intact cellular junctions to mesenchymal cells with enhanced migratory and invasive capacities. This transition is primarily orchestrated by a set of key transcription factors (EMT-TFs) to suppress the expressions of epithelial markers and enhance the expressions of mesenchymal markers. Multiple signaling pathways, such as TGF-β, Wnt, Notch, Shh, receptor tyrosine kinase, and cytokine receptor signaling pathways, collectively orchestrate the EMT process. Mature Schwann cells experience EMT-like process and switch to repair Schwann cells after peripheral nerve injury.

Schwann cells are anatomically associated with peripheral nerve roots, trunks, and terminal branches. Classified based on their anatomical location and morphology, Schwann cells are typically categorized as myelinating Schwann cells, non-myelinating Schwann cells, and terminal Schwann cells. Myelinating Schwann cells are responsible for enveloping large-diameter neuronal axons, forming multilayered myelin sheaths that are essential for rapid saltatory conduction of nerve impulses. Terminal Schwann cells surround the terminal regions of neurons and contribute to the formation of the neuromuscular junction (Stierli et al., 2019). Following peripheral nerve injury, mature Schwann cells undergo significant phenotypic changes, transform into repair Schwann cells, and facilitates nerve regeneration (Zhang et al., 2023). Emerging studies highlight the crucial role of the Schwann cell EMT-like process, which appears fundamental for their conversion to a repair phenotype and subsequent nerve regeneration (Figure 2). In this review, we systematically elucidate the molecular mechanisms underlying the EMT process, with particular focus on its activation in Schwann cells following peripheral nerve injury. Furthermore, we critically examine the pivotal role of Schwann cell EMT-like process in facilitating functional recovery of injured nerves, highlighting its significance in peripheral nerve repair and regeneration.

## Characteristics of EMT

### Cellular and molecular features of EMT

EMT is a highly dynamic activity that facilitates the conversion of epithelial cells into mesenchymal phenotypes (Figure 2). Epithelial cells are characterized by their well-organized cellular architecture, maintained through an intricate network of intercellular junctions, including tight junctions, adherens junctions, gap junctions, and desmosomes. These cellular junctions tightly pack epithelial cells in a polygonal or columnar shape with distinct apical-basal polarity (Bartle et al., 2018). Furthermore, epithelial cells are firmly anchored to the basement membrane through hemidesmosomes and α6β4 integrins, which significantly restrict their migratory capacity (Te Molder et al., 2021). The molecular signature of epithelial cells is defined by the expression of specific junctional proteins, including tight junction proteins claudin, occluding, and ZO-1 as well as adherens junction protein E-cadherin. These junctional proteins serve as canonical epithelial markers and play crucial roles in maintaining epithelial integrity and function. Other epithelial cell markers contain epithelial cell adhesion molecules, α6β4 integrins, and cytokeratins (Yang et al., 2020).

Mesenchymal cells exhibit distinct morphological and functional characteristics, featuring a spindle-shaped morphology with front-rear polarity and enhanced motility. Unlike epithelial cells, mesenchymal cells lack the tightly organized junctional complexes but instead possess vimentin-based intermediate filaments and actin stress fibers (Vallenius, 2013; Costigliola et al., 2017). Mesenchymal cells have increased extracellular matrix production capacity and establish connections with the extracellular matrix via integrin-containing focal adhesions. The molecular signature of mesenchymal cells is characterized by the expression of specific markers, including N-cadherin, vimentin, fibronectin, β1 and β3 integrins, and matrix metalloproteinases (Yang et al., 2020).

During the EMT process, cells experience cytoskeletal and extracellular matrix remodeling, the loss of apical-basal polarity and the gain of back-front polarity, the acquisition of cellular individualization, the enhancement of migratory capacity, and the development of invasive potential through basement membrane penetration (Dongre and Weinberg, 2019; Lambert and Weinberg, 2021). The complete transition from a fully epithelial state to a fully mesenchymal state is a relatively rare phenomenon. More commonly, epithelial cells undergo a partial EMT process and proceed to an epithelial/mesenchymal intermediate state. Epithelial cells can overcome the thermodynamic obstacles and switch to multiple transitional states between epithelial cells and mesenchymal cells, including a metastable intermediate state 1 with dissolution of cellular junctions and loss of apical-basal polarity, a thermodynamically and kinetically stable intermediate state 2, and a metastable intermediate state 3 with presence of residual junction puncta and acquisition of back-front polarity (Nieto et al., 2016). Cells in these transitional states display a unique hybrid phenotype, maintaining certain epithelial characteristics while acquiring some mesenchymal properties. Some transitional cells express neither E-cadherin or N-cadherin while some transitional cells co-express both cytokeratin and vimentin (Nieto et al., 2016). The hybrid epithelial-mesenchymal characteristics exhibited by these transitional cells generate substantial phenotypic heterogeneity, manifesting in varied adhesive properties and diverse migratory and invasive capacities. Consequently, the accurate determination of EMT status requires multidimensional evaluation that integrates both cellular phenotypic features and molecular characteristics. This approach should encompass assessment of cellular morphology and polarity, evaluation of migratory and invasive potential, quantification of a set of epithelial and mesenchymal markers as well as key EMT regulators, and the consideration of transitional states and partial EMT characteristics (Yang et al., 2020).

### Signaling pathways in EMT

The EMT process is regulated through multiple signaling pathways, including transforming growth factor-β (TGF-β) signaling, Wnt signaling, Notch signaling, sonic hedgehog (Shh) signaling, and signaling pathways mediated by receptor tyrosine kinases or cytokine receptors. Among these, TGF-β serves as a principal EMT inducer by binding to TGF-β type I receptor, forming and phosphorylating receptor complexes, and acitivating both canonical TGF-β/Smad signaling and non-canonical pathways, such as ERK1/2, JNK, p38 MAPK, PI3K/AKT, RhoA, and Cdc42/Rac signaling molecules (Li et al., 2017). Wnt ligands initiate signaling by binding to the frizzled receptors, forming a cell surface complex with LRP5/6 co-receptors, which subsequently recruits and activates Dishevelled proteins, leading to the stabilization and accumulation of β-catenin (Angers and Moon, 2009). Delta-like and Jagged family members bind to Notch signaling receptors, triggering the cleavage of Notch and the subsequent release of its active intracellular domain (Wang et al., 2015). Shh proteins bind to the 7-pass transmembrane protein patched and regulate the activity of downstream Gli-family transcription factors (Syed et al., 2016). Receptor tyrosine kinases and cytokine receptors, upon binding to their corresponding ligands, activates the PI3K/AKT signaling and JAK/STAT signaling pathways, respectively (Dongre and Weinberg, 2019). Many of these signaling molecules, such as SMAD2/3, β-catenin, intracellular Notch, NF-κB, and STAT3, translocate into the nucleus and turn on the expressions mesenchymal genes as well as many transcription factors that mediates the EMT process. These signaling pathways function cooperatively to drive EMT, with Notch signaling emerging as a particularly prominent driver (Derynck et al., 2014; Deshmukh et al., 2021).

### Regulators of EMT

The EMT process is finely regulated by a diverse array of transcription factors and epigenetic regulatory programs. Transcription factors are functionally critical proteins that recognize specific DNA sequences and directly modulate the transcription of target genes (Lambert et al., 2018). Transcription factors play essential roles in determining and manipulating cell fate, driving diverse cellular processes such as cell differentiation, de-differentiation, and trans-differentiation (Takahashi and Yamanaka, 2016). The EMT process represents a complex de-differentiation program regulated by an intricate network of transcriptional factors, including the Zeb family members Zeb1 and Zeb2; the Snail family members Snail1 and Snail2; the Twist family members Twist1 and Twist2; the Kruppel-like factor family members KLF4, KLF8, and KLF10; the Forkhead box family members FOXC1, FOXC2, FOXQ1, FOXK1, FOXG1, FOXF2, FOXN2, and FOXO3a; the SRY-related HMG-box family members Sox4, Sox9, and Sox11; the RUNX family members RUNX1 and RUNX2; the GATA family members GATA4, GATA6, Wilms’ tumor 1 (WT1), Goosecoid, Six1, paired-related homeobox 1 (PRRX1), Elk3, and Brachyury; the AP-1 family members FOSL1, FOSL2, OVOL1, OVOL2, and TFAP2A; as well as the E2A proteins E12 and E47 (Debnath et al., 2022). For instance, zinc finger family transcription factors Zeb1 and Zeb2 bind to the promoter of the E-cadherin coding gene CDH1, repress the expression of E-cadherin, and activate all three types of EMT, that are tissue development, fibrosis, and cancer progression (Sánchez-Tilló et al., 2010; Debnath et al., 2022; Kinouchi et al., 2024). These transcription factors prompt the induction of super-enhancers and drive cellular transition in a cooperative manner (Chang et al., 2016). Elevated expression of Zeb1 in mouse oral cancer cells increases the endogenous levels of Zeb2, and conversely, heightened Zeb2 expression similarly upregulates Zeb1 (Kinouchi et al., 2024). In mouse mammary epithelial EpH4 cells, ectopically expressed FOSL1 binds to the regulatory sequence regions of the transcription factor-coding genes Zeb1 and Zeb2, as well as to the EMT-inducer TGF-β, thereby elevating the expression levels of these transcriptional activators, activating TGF-β signaling, and functioning as a powerful driver of the EMT process (Bakiri et al., 2015). Despite these synergistic interactions, transcription factors involved in regulating EMT may exhibit diverse and sometimes opposing biological functions. In the mouse mammary gland epithelial cell line NmuMG, TFAP2A directly interacts with the promoter region of Zeb2 and upregulates Zeb2 expression; however, contrary to expectations, TFAP2A functions as an EMT suppressor and perturbs TGF-β1-induced EMT (Dimitrova et al., 2017).

The activation of the EMT process and corresponding phenotypical transitions do not inherently depend on alterations in DNA sequence but can be triggered by epigenetic modifications, such as DNA methylation and histone modifications. DNA methylation, through the transfer of a methyl group, generates 5-methylcytosine on the C5 position and regulates gene expression (Moore et al., 2013). DNA methylation is frequently observed in cells undergoing the EMT process, while cellular demethylation, achieved through the application of the DNA methylation inhibitor 5-azacytidine or the silencing of DNA methyltransferases (DNMTs), promotes the reversal of the EMT process (Galle et al., 2020). Transcriptional factors, besides their genetic functions, can also modulate gene expression at the epigenetic level. The EMT inducer ZEB1 interacts with DNMT1, enhances the 5-methylcytosine modification at the E-cadherin promoter, and consequently represses E-cadherin expression (Fukagawa et al., 2015). Histone posttranslational modifications, such as histone methylation and acetylation, play an important role in modulating genomic architecture and regulating numerous biological activities associated with EMT (Millán-Zambrano et al., 2022). Epithelial cells transition from an active state characterized by enriched histone 3 lysine 27 acetylation (H3K27ac) and histone H3 tri-methylated lysine 4 (H3K4me3) to a repressed state marked by trimethylation of histone H3 lysine 27 (H3K27me3), lysine 9 (H3K9me3), and DNA methylation as EMT progresses (Hatta et al., 2018; Segelle et al., 2022). Numerous epigenetic regulators, including histone demethylase LSD1, histone methyltransferase PRMT5, histone deacetylase HDAC1, and histone acetyltransferase CBP, directly modulate the expressions of EMT-related transcription factors and/or interact with these transcription factors to regulate the expressions of their target genes, thereby influencing the EMT process (Lu and Kang, 2019).

Non-coding RNAs, such as microRNAs (miRNAs), long non-coding RNAs (lncRNAs), and circular RNAs (circRNAs), constitute the vast majority of RNA transcripts in mammals. These non-coding RNAs control the gene expressions without altering DNA sequences and hence are also recognized as key players in the epigenetic regulatory landscape (Wei et al., 2017; Panni et al., 2020). Enforced expressions of miR-200 family members, which target and reduce the levels of ZEB1 and ZEB2 at the post-transcriptional level, inhibits TGF-β-induced EMT. Conversely, downregulation of miR-200 family members suppresses E-cadherin expression, enhances vimentin expression, and promotes the induction of EMT (Gregory et al., 2008; Park et al., 2008). ZEB1, in turn, binds to the promoter of miR-200 and suppresses its transcription, creating a reciprocal regulatory relationship (Brabletz and Brabletz, 2010). Similarly, miR-34 and Snail form a double-negative feedback loop that governs the equilibrium between the epithelial state and the mesenchymal state (Siemens et al., 2011). LncRNA CARMN inhibits the expression of matrix metalloproteinase 2 (MMP2), a protease responsible for degrading the extracellular matrix, thereby impeding the EMT process in breast cancer cells (Liao et al., 2024). LncRNA 01016 suppresses DHX9 proteasomal degradation, increases DHX9 expression, activates the PI3K/Akt signaling pathway, and improves breast cancer cell migration (Sun et al., 2023). CircRNA PTK2 enhances the stability of SETDB1 and accelerates SETDB1-induced EMT in bladder cancer (Meng et al., 2023). The interplay between these non-coding RNAs in regulating EMT has also been extensively investigated. For instance, miR-200 interacts with and inhibits lncRNA HOTAIR, thereby suppressing EMT in renal cell carcinoma (Dasgupta et al., 2018). LncRNA KB-1732A1.1 physically interacts with miR-200 and contribute to breast cancer cell EMT (Li et al., 2019). LncRNA IUR, on the other hand, enhances miR-200 expression, thereby suppressing ZEB1 expression and inhibiting pancreatic cancer cell migration and invasion (Sun et al., 2019). LncRNA XIST boosts ZEB1 expression through competitively binding to miR-429, and contributes to pancreatic cancer cell EMT (Shen et al., 2019). CircRNA circ_0001666, via the circ_0001666/miR-1251/Sox4 axis, facilitates the EMT process in pancreatic cancer (Zhang et al., 2021).

### EMT-like process of Schwann cells during peripheral nerve regeneration

**FIGURE 3:**
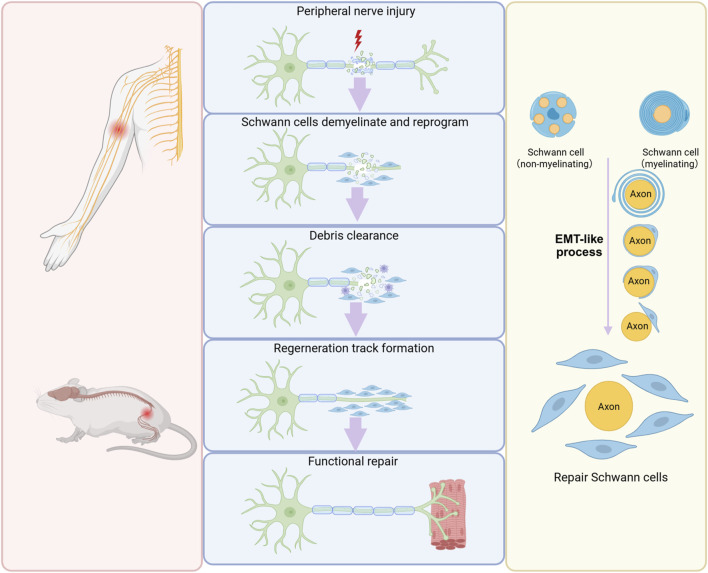
Schwann cell EMT during peripheral nerve regeneration. Following peripheral nerve injury, mature Schwann cells undergo EMT, convert into a repair-supportive phenotype, and generates a permissive microenvironment for nerve regeneration.

Tissue repair and regeneration constitute a highly orchestrated, multistage process encompassing hemostasis, inflammation, cell proliferation, and tissue remodeling. This process relies on the activation of intrinsic healing mechanisms and the enhancement of endogenous regenerative capacity. Central to this regenerative cascade is EMT, an epitome of cellular plasticity that drives cellular reprogramming during tissue repair (Valcourt et al., 2016; Lambert and Weinberg, 2021; Youssef and Nieto, 2024). Schwann cells undergo an EMT-like process essential for their plasticity, enabling a phenotypic switch from a differentiated to a repair state after peripheral nerve injury. This process is critical for creating a permissive microenvironment that promotes axonal regrowth and functional recovery (Figure 3).

### Schwann cell in homeostasis and regeneration

In intact peripheral nerves, myelinating Schwann cells are predominantly quiescent and exhibit minimal cellular turnover, typically persisting without renewal throughout the whole life period. Non-myelinating Schwann cells also display a low renewal frequency, with a remarkably long turnover time of approximately once every 72 months (Stierli et al., 2019). Following peripheral nerve injury, Schwann cells demonstrate remarkable plasticity. Damage to peripheral nerves disrupts the interaction between Schwann cells and neuronal axons, eliminates inhibitory signals from the axons, and hence enables mature Schwann cells to revert to a dedifferentiated state (Merrell and Stanger, 2016). Damaged axons also release some signal molecules, such as mitochondrial alarmins and ATP, which play a crucial role in triggering the activation of Schwann cells (Duregotti et al., 2015; Negro et al., 2016). The distinctive combination of cell de-differentiation and activation is a distinctive hallmark of adaptive reprogramming and indicates that Schwann cells switch to a repair-supportive phenotype designated as repair Schwann cells after nerve injury (Jessen and Mirsky, 2016; Zhao et al., 2024). Lineage tracing studies have revealed that both myelinating and non-myelinating Schwann cells undergo a phenotypic transition into repair Schwann cells characterized by extended cytoplasmic processes (Gomez-Sanchez et al., 2017). These repair Schwann cells actively participate in myelin clearance by phagocytosing and degrading myelin sheath debris, thereby eliminating inhibitory factors that impede axonal elongation. Furthermore, repair Schwann cells contribute to the regenerative microenvironment by secreting a spectrum of neurotrophic factors, surface proteins, and cytokines that collectively promote axonal growth. This secretory profile enables repair Schwann cells to provide essential pro-regenerative signals from the peripheral environment, supporting neuronal survival and facilitating nerve regeneration. Importantly, the morphological transformation of mature Schwann cells into an elongated bipolar configuration facilitates the organization of aligned guidance structures known as Bungner’s bands (Jessen and Mirsky, 2016).

Following the successful regrowth of injured axons, repair Schwann cells undergo a series of critical morphological and functional changes. Schwann cells establish direct contact with regenerating neuronal axons, initiate a process of radial contraction that significantly reduces their elongated morphology, and undergo re-differentiation to their mature phenotype. This maturation process culminates in the re-establishment of the characteristic Schwann cell-axon relationship, where the re-differentiated Schwann cells envelop the regenerated axons, restoring the normal structural organization of the nerve fiber (Jessen and Mirsky, 2016).

### EMT-like transition of Schwann cells following peripheral nerve injury

Mature Schwann cells possess several epithelial-like characteristics, including cellular polarity, the presence of intercellular junctions, and the expression of epithelial cell markers (Bunge et al., 1986). A key event in the transformation of mature Schwann cells into repair Schwann cells is the activation of the EMT-like process. Transcriptomic profiling of sorted and purified Schwann cells collected from mouse distal nerve stumps at 6 days post sciatic nerve transection reveals significant downregulation of Cdh1, the gene encoding the epithelial marker E-cadherin, compared to Schwann cells from intact sciatic nerves. Concurrently, there is a marked enrichment of an EMT-related gene set and a Yamanaka reprogramming factor Myc gene set, indicating the dedifferentiation of mature Schwann cells at the injured distal nerve stumps. Further comparison of transcriptomes between Schwann cells within the nerve bridge and those at the distal nerve stumps demonstrates that bridge Schwann cells exhibit greater proliferative capacity, heightened TGF-β signaling activity, and more pronounced mesenchymal characteristics. The robust activation of TGF-β signaling within the nerve bridge drives Schwann cell reprogramming, enhances their organization and sorting, and facilitates the creation of a regeneration-permissive microenvironment (Clements et al., 2017). Similarly, RNA sequencing of mouse distal nerve stumps at 7 days post sciatic nerve injury reveals a reduction in Cdh1 expression alongside the upregulation of multiple EMT-promoting genes, including Tgfb1, Shh, Fosl1, and tenascin-C-coding gene Tnc (Arthur-Farraj et al., 2017). Additionally, a significant number of EMT-associated miRNAs are differentially expressed in mouse injured distal nerve stumps, further underscoring the role of EMT-like process in Schwann cell reprogramming and peripheral nerve regeneration (Viader et al., 2011; Arthur-Farraj et al., 2017).

The activation of EMT-like process and the phenotypic switch of Schwann cells are also observed in rats subjected to peripheral nerve injury, demonstrating a high degree of conservation in the injury response mechanisms of Schwann cells across species. In the distal nerve stumps of rats following surgical removal of a 10-mm sciatic nerve segment, genes encoding several MMPs, including Mmp7, Mmp9, and Mmp12, are significantly up-regulated. This upregulation suggests extensive remodeling of the extracellular matrix, which may facilitate the acquisition of migratory capabilities by Schwann cells, a critical step in the nerve repair process (Yu et al., 2016). Consistent with sequencing data from injured mouse sciatic nerves, transcriptomic analysis of immunopanned Schwann cells purified from rat sciatic nerves following nerve crush reveals the upregulation of EMT enhancer Tnc as well as significant changes in the expressions of extracellular matrix-related genes (Brosius Lutz et al., 2022). These findings, by eliminating confounding factors introduced by heterogeneous cell populations in peripheral nerves, provide direct evidence of transcriptomic changes occurring in purified Schwann cells after nerve injury and demonstrate the alternation of EMT-associated genes. And these observations demonstrate that the EMT-like transition in Schwann cells is a conserved response, occurring after both relatively mild crush injuries and more severe injuries, for instance, nerve transition and long distance nerve defect. The activation of Schwann cell EMT-like program parallels the wound healing processes observed in other regenerative tissues and is biologically advantageous, as it facilitates successful nerve regeneration by promoting Schwann cell migration, extracellular matrix remodeling, and the creation of a pro-regenerative microenvironment (Jessen and Arthur-Farraj, 2019).

### Targeting EMT-like process of Schwann cells in the treatment of peripheral nerve injury

It is worth noting that although the peripheral nervous system has regeneration capacity, the self-regeneration of injured peripheral nerves is often inadequate and unsatisfactory due to limited speed of nerve regrowth (Avraham et al., 2021). Under some circumstances, such as serious peripheral nerve injury with long nerve gaps and nerve injury in aged individuals, the regeneration speed of injured nerves may be further diminished (Yi et al., 2019). This can result in chronic denervation of target tissues and organs, the atrophy of limb muscle, and ultimately, failure of functional recovery. Therapeutic strategies that enhance the EMT-like process in Schwann cells and sustain their repair state are essential for accelerating axonal regeneration and improving functional recovery after nerve injury. By promoting this transition, these approaches facilitate a pro-regenerative microenvironment, enhance Schwann cell migration, and support the structural and functional restoration of damaged nerves.

The biological involvement of TGF-β signaling in peripheral nerve injury and regeneration is well-established (Li et al., 2017; Ding et al., 2024; Lee et al., 2024). In response to injury signals, TGF-β is secreted by Schwann cells and other cell populations within peripheral nerves, such as macrophages and fibroblasts, leading to its elevated expression in the wound microenvironment (Ye et al., 2022). Experimental treatment of Schwann cells with recombinant TGF-β protein results in the downregulation of key myelin-related molecules, such as P0, MBP, and PMP22, and inhibits Schwann cell differentiation and myelination processes (Guénard et al., 1995; Awatramani et al., 2002). Morphologically, treatment with recombinant TGF-β protein disrupts gap junction-mediated intercellular coupling, induces a transition in Schwann cells to a flattened, multipolar morphology, and promotes the formation of bands of Büngner (Chandross et al., 1995; Ribeiro-Resende et al., 2009). Functionally, TGF-β levels critically regulate key regenerative behaviors of Schwann cells. Silencing TGF-β1 suppresses both proliferation and apoptosis of Schwann cells, whereas overexpression of TGF-β1 stimulates both processes (Li et al., 2015). Complementary to these findings, exogenous TGF-β treatment increases the mRNA and protein expressions of MMP2 and MMP9 and largely enhances Schwann cell migration and invasion (Muscella et al., 2020). The pro-migratory effect of TGF-β is biologically consistent with the biological functions of many MMPs, as evidenced by direct studies showing MMP7 and MMP9 promotes Schwann cell migration in rat sciatic nerve injury models (Wang et al., 2019; Lu et al., 2022). These findings collectively demonstrate that TGF-β effectively stimulates an EMT-like process in Schwann cells across morphological, functional, and molecular dimensions. Still, many aspects of this TGF-β-induced reprogramming remain unresolved, such as epigenetic regulatory mechanisms and non-coding RNA networks. Moreover, whether TGF-β drives a full or partial EMT-like transition of Schwann cells and the persistence of EMT-like transformation remain unexamined. The therapeutic benefits of exogenous TGF-β in treating peripheral nerve injury have been consistently validated in various animal models. For instance, in a study involving dogs with a 50-mm sciatic nerve gap, the combination of TGF-β with autologous adipose-derived mesenchymal stem cells and xenogeneic acellular nerve matrix grafts yielded significantly superior repair outcomes compared to the use of autologous adipose-derived mesenchymal stem cells and xenogeneic acellular nerve matrix graft alone (Luo et al., 2012). Beyond its efficacy in treating long nerve gaps, TGF-β has also shown promise in the repair of chronically injured nerves. Studies demonstrate that both standalone TGF-β treatment and combined therapy with TGF-β plus forskolin effectively reactivate Schwann cells, significantly enhancing axonal growth and promoting nerve repair (Sulaiman and Gordon, 2002; Sulaiman and Gordon, 2009; Sulaiman and Dreesen, 2014; Sulaiman et al., 2018). These findings underscore the versatility of TGF-β as a powerful therapeutic agent, capable of addressing severe peripheral nerve injuries as well as chronic peripheral nerve injuries by rejuvenating Schwann cell function and creating a pro-regenerative microenvironment.

Beyond TGF-β, other members of the TGF-β superfamily critically modulate Schwann cell behavior. Bone morphogenetic protein 7 (BMP-7) significantly supports Schwann cell proliferation (Kokubu et al., 2018), while activin A enhances both proliferation rate and migration ability of Schwann cells (Li et al., 2022). In addition to TGF-β family members, other signaling molecules and regulatory factors play critical roles in modulating Schwann cell phenotype and plasticity. PKCε, a calcium-independent protein kinase, is expressed in both myelinating and non-myelinating Schwann cells, existing in both phosphorylated and non-phosphorylated states. Treatment with the PKCε activator dicyclopropyl-linoleic acid does not affect Schwann cell viability but significantly enhances the proliferation and migration of Schwann cells. Furthermore, PKCε activation reduces the expression of E-cadherin, raises the expressions of N-cadherin and EMT-related transcription factor Snail, and induces the cytoskeleton rearrangement and morphological change. These observations collectively indicate that PKCε activation promotes EMT-like process of Schwann cells, highlighting its role in regulating Schwann cell plasticity and supporting nerve repair processes (Mohamed et al., 2023). FOSL1, a transcription factor known to drive the EMT process (Casalino et al., 2023), has been identified as to be significantly upregulated in Schwann cells at injury sites following peripheral nerve injury (Chen et al., 2023a). Functional studies reveal that Schwann cells overexpressing FOSL1 via lentiviral infection exhibit markedly enhanced migratory capacity. Conversely, Schwann cells transfected with siRNA targeting FOSL1 show compromised proliferation rates and slower migration speeds. *In vivo* experiments further demonstrate that local injection of siRNA targeting FOSL1 at the injury site in rats subjected to sciatic nerve crush impairs Schwann cell activity, retards axonal regrowth, and delays the remyelination of elongated axons as well as the recovery of nerve conductance and nerve function (Chen et al., 2023a). Similarly, runt-related transcription factor 2 (Runx2), a transcription factor activating EMT pathway (Liu et al., 2020), shows increased local expression in the injured peripheral nerves (He et al., 2025). Elevated Runx2 activates the stemness factor Sox2, drives the morphological transition of Schwann cells from a spindle-shaped to a flat, rounded phenotype, and promotes EMT-like process in Schwann cells (He et al., 2025). Following peripheral nerve injury, the expression of the microphthalmia-associated transcription factor (MITF) is upregulated through post-transcriptional mechanisms and MITF shuffles from the Schwann cell cytoplasm to the nucleus to execute its transcriptional function. Mutations in MITF increase the adhesion properties of Schwann cells, impair their migratory ability, and disturb proper Schwann cell dedifferentiation after nerve injury. These defects ultimately hinder axonal regrowth and impede nerve regeneration (Daboussi et al., 2023).

Complementing EMT-associated transcription factors, the biological functions of many EMT-related non-coding RNAs in Schwann cells have been explored. For example, miR-34 has been shown to inhibit Schwann cell dedifferentiation and proliferation, while miR-200 suppresses Schwann cell migration (Viader et al., 2011; Chen et al., 2023b). These findings highlight the complex regulatory networks involving non-coding RNAs that fine-tune Schwann cell responses to nerve injury and reflect that Schwann cells can be activated to expedite nerve regeneration by modulating the expressions of these non-coding RNAs.

Given the demonstrated efficacy of EMT inducers in driving Schwann cell reprogramming, therapeutic strategies targeting EMT-like process are emerging as promising approaches for nerve regeneration. These strategies may include pharmacological activators of key signaling pathways or genetic modifications of EMT regulators. A representative example of such an EMT-like promoting agent is isoviolanthin, a natural compound isolated from dendrobium officinale. Isoviolanthin enhance Schwann cell viability and mobility via up-regulating vimentin, thereby modulating the EMT-like process. These effects demonstrate the potential of isoviolanthin to address impaired peripheral nerve regeneration (Su et al., 2025). And such interventions hold significant potential not only for treating peripheral nerve injuries but also for addressing other neurological disorders associated with Schwann cell plasticity.

While our current review article focuses on the EMT-like process of Schwann cells following peripheral nerve injury and during nerve regeneration, emerging studies demonstrate the essential involvement of Schwann cells in cancer progression, a process that share many common biological features with regeneration (Gracia et al., 2025; Zhang et al., 2025). Schwann cells may generate a specialized tumor microenvironment via a type 3 EMT-like program, and induces the invasiveness of cancer cells (Yurteri et al., 2022). For instance, dietary palmitic acid uptake activates intratumoural Schwann cells, switches Schwann cells to a pro-regenerative state, and expedites metastasis in oral carcinomas and melanoma (Pascual et al., 2021). The dual role of Schwann cells, driven by EMT-like process, in nerve regeneration and cancer progression underscores the critical need to balance their essential pro-migratory function in repair against the requirement to inhibit their pro-tumorigenic activity. Considering that EMT-mediated migration drives both beneficial repair and detrimental pathological processes like invasion and metastasis, when developing Schwann cell-based regenerative therapies, their pro-tumorigenic potential via EMT-like process must be rigorously considered. Future research is needed to delineate the distinct molecular mechanisms, spatiotemporal regulation, and microenvironmental cues that differentiate physiological regeneration from pathological invasion. Identifying these differences is fundamental for designing precise therapeutic strategies that effectively promote tissue repair while avoiding the inadvertent stimulation of cancer progression.

## Concluding remarks

A key factor contributing to the superior regenerative capacity of peripheral nerves compared to central nerves is the activation of EMT-like process in Schwann cells. Following peripheral nerve injury, Schwann cells initiate EMT-like program, switch to a plastic repair state, and contribute to the generation of a permissive microenvironment for axonal elongation and nerve repair. This article provides insight into the cellular and molecular hallmarks of EMT, emphasizing its critical role in Schwann cell plasticity and its contribution to successful peripheral nerve regeneration.

Notably, while the de-differentiation of mature Schwann cells is crucial for tissue remodeling, the subsequent re-differentiation of repair Schwann cells and their wrapping around regenerated axons are equally vital for achieving functional recovery. Prolonged maintenance of the EMT-like state may impede Schwann cell re-differentiation, thereby negatively impacting nerve regeneration. For instance, Schwann cells deficient in the EMT inducer Zeb2 exhibit sustained de-differentiation but fail to remyelinate effectively, resulting in compromised nerve regeneration (Quintes et al., 2016; Brinkmann and Quintes, 2017). In addition, excessive or prolonged EMT-like process can lead to neurofibrosis (Wu et al., 2023). Successful nerve regeneration requires precise temporal regulation of the EMT-like process in Schwann cells. Hence, a balanced approach is essential, ensuring robust de-differentiation during the early phases of injury to facilitate tissue repair, followed by timely re-differentiation in later stages to support remyelination and functional restoration.

## Abbreviations

EMT, epithelial-mesenchymal transition; TGF-β, transforming growth factor-β; Shh, sonic hedgehog; WT1, Wilms’ tumor 1; PRRX1, paired-related homeobox 1; DNMTs, DNA methyltransferases; H3K27ac, histone 3 lysine 27 acetylation; H3K4me3, histone H3 lysine 4 trimethylation; H3K27me3, histone H3 lysine 27 trimethylation; H3K9me3, histone H3 lysine 9 trimethylation; miRNA, microRNA; lncRNA, long non-coding RNA; circRNA, circular RNA; MMP, matrix metalloproteinase; MITF, microphthalmia-associated transcription factor.
